# Liquid biopsy: an examination of platelet RNA obtained from head and neck squamous cell carcinoma patients for predictive molecular tumor markers

**DOI:** 10.37349/etat.2023.00143

**Published:** 2023-06-30

**Authors:** Lisa T. Huber, Johann M. Kraus, Jasmin Ezić, Amin Wanli, Marco Groth, Simon Laban, Thomas K. Hoffmann, Barbara Wollenberg, Hans A. Kestler, Cornelia Brunner

**Affiliations:** University of Campania “Luigi Vanvitelli”, Italy; ^1^Department of Oto-Rhino-Laryngology, Head and Neck Surgery, Ulm University Medical Center, 89075 Ulm, Germany; ^2^Institute of Medical Systems Biology, Ulm University, 89081 Ulm, Germany; ^3^Leibniz Institute of Aging – Fritz Lipmann Institute, CF DNA sequencing, 07745 Jena, Germany; ^4^Clinic for Otorhinolaryngology, Head and Neck Surgery, Technical University of Munich, 80333 Munich, Germany

**Keywords:** Tumor educated platelets, liquid biopsy, head and neck

## Abstract

**Aim:**

Recently, a tumor cell-platelet interaction was identified in different tumor entities, resulting in a transfer of tumor-derived RNA into platelets, named further “tumor-educated platelets (TEP)”. The present pilot study aims to investigate whether such a tumor-platelet transfer of RNA occurs also in patients suffering from head and neck squamous cell carcinoma (HNSCC).

**Methods:**

Sequencing analysis of RNA derived from platelets of tumor patients (TPs) and healthy donors (HDs) were performed. Subsequently, quantitative reverse transcription-polymerase chain reaction (qRT-PCR) was used for verification of differentially expressed genes in platelets from TPs and HDs in a second cohort of patients and HDs. Data were analyzed by applying bioinformatic tools.

**Results:**

Sequencing of RNA derived from the tumor as well as from platelets of TPs and HDs revealed 426 significantly differentially existing RNA, at which 406 RNA were more and 20 RNA less abundant in platelets from TPs in comparison to that of HDs. In TPs’ platelets, abundantly existing RNA coding for 49 genes were detected, characteristically expressed in epithelial cells and RNA, the products of which are involved in tumor progression. Applying bioinformatic tools and verification on a second TP/HD cohort, collagen type I alpha 1 chain (COL1A1) and zinc finger protein 750 (ZNF750) were identified as the strongest potentially platelet-RNA-sequencing (RNA-seq)-based biomarkers for HNSCC.

**Conclusions:**

These results indicate a transfer of tumor-derived messenger RNA (mRNA) into platelets of HNSCC patients. Therefore, analyses of a patient’s platelet RNA could be an efficient option for liquid biopsy in order to diagnose HNSCC or to monitor tumorigenesis as well as therapeutic responses at any time and in real time.

## Introduction

Head and neck squamous cell carcinoma (HNSCC) describes tumors that originate from the epithelium of the oral cavity, pharynx, and larynx. They represent the sixth most prevalent cancer type globally with 932,000 cases and a mortality of 467,000 deaths every year as of 2020 [1]. Risk factors primarily include smoking and excessive alcohol consumption, as well as aging, diet, oral hygiene, and family history. Additionally, infection with human papilloma virus (HPV), a circular DNA virus that targets the epithelial cells of the mucosa, plays an important role in the development of HNSCC. In general, treatment options include surgery, radio-, chemo- and immunotherapy [2]. Besides this, novel therapeutic approaches based on molecular tumor markers were developed like the targeting of epidermal growth factor receptor (EGFR), a highly upregulated receptor in HNSCC associated with poor prognosis. Additionally, as an immunotherapeutic approach, the checkpoint inhibitor pembrolizumab has been approved by the Food and Drug Administration (FDA) in 2019 as a first-line treatment for metastatic or recurrent HNSCC [3]. Despite recent advances in HNSCC therapeutic options, the mortality rate is still around 50%, which indicates the need for more effective therapies and diagnostic as well as disease monitoring tools. For this, reliable biomarkers should be defined that can predict the onset and progression of HNSCC or even the therapeutic outcome. Moreover, based on biomarker expression personalized therapeutic options could be developed and applied. However, presumably because of the high heterogeneity in HNSCC, in contrast to other tumor entities, a robust biomarker panel for HNSCC has not been defined so far, as applicable in clinical settings [4].

There are different ways to obtain information about the genetic changes of the tumor. Although regarded as the gold standard for tumor diagnosis, tissue biopsy suffers from several limitations. Firstly, tissue biopsy is invasive in nature and can potentially lead to complications, causing limitations in the reproducibility and repeatability of the diagnosis. On top of that, tumor heterogeneity impedes the ability to obtain spatially and temporally accurate information about the tumors [5]. For this reason, recent efforts have been made to find new methods for cancer diagnosis and screening options.

Liquid biopsy describes the analysis of biomarkers released by tumors into body fluids, such as blood, urine, or saliva. These biomarkers could be cells, nucleic acids, or proteins. Liquid biopsy offers a minimally invasive procedure, which is more easily applicable and could be repeated more frequently, offering a more real-time and dynamic snapshot of the tumor, which combats the spatial and temporal limitation of tissue biopsy [6]. This enables clinicians not only to utilize liquid biopsy as an effective diagnostic tool but also as an instrument for therapy and tumor progression monitoring. The advantage of analyzing genetic markers also offers a potential approach for personalized treatment [7].

Information about potentially existing tumors could be obtained from several sources through liquid biopsy, like circulating tumor cells (CTCs), circulating cell-free tumor DNA (ctDNA), and exosomes [8–10]. Their analyses were suggested as sensitive and specific for cancer prediction, diagnosis, prognosis, and monitoring over time [11–13] including HNSCC [14–18]. Thus, a recent study on 21 patients with locally advanced oropharyngeal carcinoma treated with neoadjuvant chemotherapy suggested that the variation of CTC numbers might reflect the effectiveness of treatment [19]. Also, the potential of ctDNA as a biomarker for detecting minimal residual disease and recurrence in HNSCC was demonstrated recently [20]. Additionally, the analyses of circulating exosomes emerged as a potential tool for HNSCC diagnosis [21] and several studies correlated proteins identified in patients’ exosomes with the activity of tumor disease and therapy responses [21, 22].

However, with regard to head and neck tumors, all these types of liquid biopsies are far from routine clinical use because, despite many advantages over standard diagnostics, they have some limitations. Over the last years, another form of liquid biopsy has received considerable attention—the analyses of so-called “tumor-educated platelets (TEP)”. Platelets interact with tumor cells leading to platelet degranulation, known as tumor cell-induced platelet aggregation. The release of their storage products supports tumor growth, angiogenesis, and tumor spread [23].

Cancer-platelet interaction is a two-way communication path. Not only does platelet activation promote tumor progression, but also tumor cells affect platelets, specifically, their RNA profile. The idea of platelets having a distinct RNA profile in cancer patients compared with healthy controls was first introduced in 2010 [24]. In this study, Calverley et al. [24] demonstrated that platelets of metastatic lung cancer patients exhibit 200 differentially expressed genes compared to healthy controls. In 2011, Nilsson et al. [25] described the transfer of tumor cell RNA into platelets, leading to the formation of so-called TEP. Based on platelet RNA profiles of 283 patients, Best et al. [26] developed an algorithm that distinguished healthy and tumor patients (TPs) with an accuracy of 96%. In this study, six tumor entities (non-small cell lung cancer, colorectal carcinoma, breast carcinoma, hepatobiliary carcinoma, glioblastoma, and pancreatic carcinoma) have been differentiated.

In recent years, great efforts have been made to define TEP-derived biomarkers for the diagnosis of a wide variety of tumors for different cancer entities. Thus, applying particle-swarm optimization-enhanced algorithms, Best et al. [27] defined a biomarker panel allowing the detection of early- and late-stage non-small cell lung cancer with an accuracy of 81% and 88%, respectively. Sheng et al. [28] identified a biomarker panel of 48 genes for the diagnostic of the same tumor entity with an accuracy of 89%, whereas Xing et al. [29] identified ITGA2B as a diagnostic and prognostic TEP-based marker for non-small cell lung cancer, just to mention some of the TEP-RNA-sequencing (RNA-seq) work on this cancer entity. Additionally, TEP-derived RNA panels, determined again by swarm intelligence, were applied to identify glioblastoma patients with an accuracy of 95% [30]. TEP-RNA-seq-derived biomarkers were also identified for sarcoma [31], colorectal [32], and liver cancer [33–36]. Moreover, various clinical studies have confirmed the analysis of TEP as a robust form of liquid biopsy and other clinical trials are currently recruiting. The study described here was initiated because of the advantages of the analysis of TEP compared to other forms of liquid biopsy and the lack of data on TEP in HNSCC.

The present work represents a pilot study with the aim to investigate whether the analysis of platelet RNA is a diagnostic option also for HNSCC. Since differentially existing messenger RNA (mRNA) in platelets of HNSCC patients were observed, the present study provides the basis for designing further prospective studies considering cohort size, robust inclusion criteria, in- or exclusion of certain tumor sites as well as HPV status.

## Materials and methods

### Patients

The study was approved by the local ethics committee of Ulm University [90/15], and written signed informed consent was obtained from all the participants prior to conducting the study. The patient characteristics of cohorts 1 and 2 are shown in Tables 1 and 2. The patients were selected based on their diagnosis. In order to achieve the most robust results possible, which are not influenced by age, gender, or side diagnoses, these data were collected, but they did not influence inclusion in this study. The only inclusion criterion as a TP was the diagnosis of HNSCC; the inclusion criterion as a healthy donor (HD) was the confirmed exclusion of a tumor disease on the day of the blood donation. For the RNA-seq experimental study design, the suggestions of Lin et al. [37] for cohort estimation were followed. In order to determine sample sizes for the pilot data, a sample size estimation based on the assumption of *d* = 2, *alpha* = 0.05, *beta* = 0.8 using a Wilcoxon-test (software: R 3.2.2) [38] was performed.

**Table 1:** TP’s and HD’s characteristics, cohort 1

**ID**	**Age (years)**	**Sex**	**Side diagnoses**	**Notes**	**Tumor localisation**	**TNM**	**HPV status**
TP_1	58	Male	None	40 py, 1 beer/day	Tongue	pT2pN0 cM0	Negative
TP_2	18	Male	None	None	Tongue	pT2pN0 cM0	Negative
TP_3	61	Male	None	40 py, 2 beers/day	Glossotonsillar	pT2pN0 cM0	Negative
TP_4	75	Male	Type 2 diabetes mellitus	None	Tongue	pT1pN0 cM0	Negative
TP_5	67	Male	Loss of sensorineural hearing	2 Beers/day	Tongue	pT3pN0 cM0	Negative
HD_1	59	Male	None	42 py	-	-	-
HD_2	22	Male	None	N.d.	-	-	-
HD_3	63	Male	Prostate adenoma, rectal adenoma, hypertension, obstructive sleep	N.d.	-	-	-
HD_4	74	Male	Apnea, loss of sensorineural hearing	None	-	-	-
HD_5	69	Male	Polyarthritis, hypertension	N.d.	-	-	-

ID: identification; py: pack years; TNM: tumor, nodes, metastasis; p: pathological; c: clinical; N.d.: no data; -: not applicable

**Table 2:** TP’s and HD’s characteristics, cohort 2

**ID**	**Age (years)**	**Sex**	**Side diagnoses**	**Notes**	**Tumor localisation**	**TNM**	**HPV status**
TP_6	68	Male	None	15 py, 1 bottle of wine/day	Oral cavity	pT1pN0 cM0	Negative
TP_7	71	Male	None	None	Tongue	pT2pN0 cM0	Negative
TP_8	61	Male	None	4 py, 2 beers/day	Oral cavity/oropharynx	pT2pN0 cM0	HPV 33
TP_9	57	Male	None	30 py, 5 beers/day	Oral cavity/tonsil	pT3pN0 cM0	Negative
TP_10	51	Female	None	1 py	Tongue	pT3pN1 cM0	Negative
TP_11	59	Male	Benign prostate hyperplasia	45 py	Tongue	pT3pN0 cM0	Negative
HD_6	28	Male	None	None	-	-	-
HD_7	28	Male	None	None	-	-	-
HD_8	71	Male	Chronic otitis media, presbyakusis, hypertension	None	-	-	-
HD_9	52	Female	Hypothyroidism	None	-	-	-
HD_10	70	Male	None	None	-	-	-
HD_11	74	Female	Loss of sensorineural hearing	None	-	-	-
HD_12	22	Male	Chronic tonsillitis	1.5 py, occasional alcohol consumption	-	-	-

-: not applicable

### Blood collection and platelets isolation

Blood (50 mL) was taken using 10 mL S-monovets (Sarstedt, Nürnbrecht, Germany) each with 3.2% citrate, from HDs and HNSCC TPs before the onset of any therapeutic measures. “Healthy” is defined here as “no diagnosed tumor” at the time of blood collection. Additionally, tissue material from the primary tumor was collected. Donors’ monovets were centrifuged at 120 × g for 20 min. The exact number of platelets that were obtained has not been determined routinely. During the examination of the platelets under the microscope, random samples showed regular platelet counts, from 150,000/µL to 450,000/µL blood. The platelet-rich plasma was centrifuged at 360 × g for 20 min. The resulting pellet was resuspended in 400 μL to 500 μL RNAlater^TM^ (Qiagen, Hilden, Sweden). To check the purity of the platelets, 10 μL of platelet suspension was diluted with 90 μL of Tuerk solution (Merck, Darmstadt, Germany) and checked microscopically. The preparation was defined as pure if at most 5 nuclear-containing cells per 10 million platelets could be identified.

### RNA isolation and RNA-seq

For isolation of total RNA 200 μL of platelet suspension was used and centrifuged at 8,000 × g for 5 min. Total RNA from platelets and tumor tissue (80 mg) was isolated according to the protocol of the mirVana™ miRNA Isolation Kit (Thermo Fisher Scientific, Waltham, US). The pieces of tissue were lysed for 4 min at 50 Hz in the TissueLyser LT (Qiagen, Hilden, Sweden) using 5 mm beads. RNA-seq was performed using the next-generation sequencing technology of Illumina (US) [39]. Library preparation was carried out with the TruSeq Stranded mRNA Kit (illumina^®^, San Diego, US) according to the manufacturer’s instructions [40]. The quality of the sequencing libraries was controlled using the Agilent 2100 Bioanalyzer (Agilent Technologies, Santa Clara, US) and the Agilent DNA 7500 Kit (Agilent Technologies, Santa Clara, US). Sequencing was performed on a Illumina Hiseq2500 sequencing system (illumina, San Diego, US) in 51 bp, single-end sequencing, and high-output mode. Fifteen samples were sequenced on 4 lanes and reached 50 ± 5 mio reads per sample. The data were extracted with the bcl2fastq conversion software v1.8.4 program (illumina^®^, San Diego, US).

### RNA-seq data analysis

Obtained reads were aligned with the human genome version hg 19 using the Bowtie2 software and the standard parameters [41]. Read counts were determined with high-throughput sequencing (HTSeq)-count based on annotation GRCh37.75 [42]. Data analysis was performed in R (4.0.2) using the integrated development environment (IDE) Rstudio (1.3.1056). Normalization and analysis of differentially existing RNAs were performed using DESeq2 (1.16.0.), and the shrinkage estimator used was “apeglm” [43, 44]. Principal component analysis (PCA) was computed within DESeq2. Clustering in the heatmaps was done using hierarchical clustering within the heatmap (1.0.12) package. For gene ontology, gene set enrichment analysis (GSEA) was performed [45–47]. For the computation of distance matrices, Euclidean distance was used. Genes with a false discovery rate (FDR) < 0.05 were considered differentially expressed.

### Quantitative reverse transcription-polymerase chain reaction

Fifty ng RNA of the platelets was used in a one-step-polymerase chain reaction (PCR) reaction using QuantiNova RT-PCR kit (ID: 208352; Qiagen, Hilden, Germany) according to the protocol of the producer. Reactions were running at the LightCycler^®^ 96 System (Roche, Basel, Schweiz). Primers were obtained from biomers.net GmbH (Ulm, Germany) and are presented in Table 3. Data were analyzed by the formula of relative quantification according to Pfaffl [48]. For statistical analyses, the Welch’s *t*-test or Mann-Whitney-*U* test was used, depending on whether the data were normally distributed or not.

**Table 3:** Primer characteristics

**Gene name/NCBI accession NR**	**Efficiency test mRNA**	**Amplicon size (bp)**	**Primer forward**	**Primer reverse**
*GAPDH*/NM_001357943.2	2.00/universal RNA	115	GCTCTCTGCTCCTCCTGTTC	ACGACCAAATCCGTTGACTC
*UBC*/NM_021009.7	2.00/universal RNA	105	CTGATCAGCAGAGGTTGATCTTT	GACGGAGTACCAGGTGCAAG
*HPRT1*/NM_000194.3	1.98/universal RNA	94	TGACACTGGCAAAACAATGCA	GGTCCTTTTCACCAGCAAGCT
*PGK1*/NM_000291.4	2.00/universal RNA	181	GGACAATGGAGCCAAGTCGG	TGGGTTGGCACAGGCTTTCT
*COL1A1*/NM_000088.4	1.96/universal RNA	185	TGGTTCGTGACCGTGACCTC	CAGGTTGCAGCCTTGGTTGG
*ZNF750*/NM_024702.3	2.00/UDSCC5	83	GCACAGAATGCCTACCTGCC	AGGAGGGTCTCCGTTCACAAC
*MAGEA6*/NM_005363.5	2.00/universal RNA	182	TCGGGGATCCCAAGAAGCTG	AATGCGAGGTCCTCCACTGA
*Ly6D*/NM_003695.3	1.89/UDSCC5	77	GCCAGCTCTCGCTTCTGCAA	TCCGCACAGTCCTTCTTCACC
*WNT5a*/XM_011534089.2	2.00/universal RNA	66	AGCGGGTTCCTGAGTGAAT	CCCCTGACCCCTCTCTAGTT
*FAT1*/NM_005245.4	2.00/universal RNA	153	ATCCTGCAACCGGCTCTCTC	CGTGGTCATTCGTGTCGCTG
*KRT5*/NM_000424.4	2.00/universal RNA	91	GCAGATCAAGACCCTCAACAAT	CCACTTGGTGTCCAGAACCT
*EGFR*/NM_005228.5	2.00/universal RNA	200	TCCTCATTGCCCTCAACACAGT	GGCAGGGTTGTTGCTGAACC
*PDPN*/NM_198389.2	1.95/universal RNA	176	CTCAAACGTGGCCACCAGTC	GCCTTCCCGACATTTTTCGCA
*CDH11*/NM_001330576.2	2.00/universal RNA	195	ACACGGCCAATGGACCAAGA	TGCTACTCATGGGCGGGATG
*CA12*/NM_206925.3	2.00/universal RNA	173	AGTTTTCCGAAACCCCGTGC	GGATGATGCCTTGGGAGAAGGA
*RBP1*/NM_001130992.3	2.00/universal RNA	161	GCTCCAGTCACTCCCCGAAA	CACCGTCCTGCACGATCTCT
*PLA2G4E*/XM_011521238.2, XM_011521239.2, NM_001206670.1, XM_011521238.2	2.00/universal RNA UDSCC5	76, 143, 193, 197	GCCAGCTACATCACCGGTCT, TGTCAACTCCAGCTACCCGC, CAGAGCCCACAAACGGATGA, TGCAGAAGCGGAAGGTCGT	TGGAGGACCAGTCAGGGTCA, TGGGGAAGGGGATGTTCTGC, CAGCCTGCCGGACATTTTTCA, ACCGGTGATGTAGCTGGCAC

NR: number; *GAPDH*: glyceraldehyde-3-phosphate dehydrogenase; *UBC*: ubiquitin C; *HPRT1*: hypoxanthine phosphoribosyltransferase 1; *PGK1*: phosphoglycerate kinase 1; *COL1A1*: collagen type I alpha 1 chain; *ZNF750*: zinc finger protein 750; *MAGEA6*: MAGE family member A6; *Ly6D*: lymphocyte antigen 6 family member D; *WNT5a*: Wnt family member 5a; *FAT1*: FAT atypical cadherin 1; *KRT5*: keratin 5; *PDPN*: podoplanin; *CDH11*: cadherin 11; *CA12*: carbonic anhydrase 12; *RBP1*: retinol binding protein 1; *PLA2G4E*: phospholipase A2 group IVE

## Results

### RNA-seq data gaining

In order to investigate whether HNSCC-derived platelets harbor RNA originally synthesized in tumor cells, RNA-seq was performed on RNA isolated from platelets of HDs or patients suffering from HNSCC. Patients and HD characteristics are depicted in Tables 1 and 2.

Additionally, RNA derived from the corresponding tumor was sequenced. Each sequenced sample showed about 50 ± 5 million reads (read length 51 bp) and 94% of the bases reached a Phred-scaled quality score Q of 30 to 40. Per sample, around 30 million reads could be mapped to genes in the human genome. More than 90% of these reads were localized in exons.

To verify the extraction of platelet RNA, platelet markers like beta 2-microglobulin (B2M), pro-platelet basic protein (PPBP), thymosin beta 4 X-linked (TMSB4X) and platelet factor 4 (PF4) could be detected in both groups—platelets from TPs as well as from HDs (Table 4).

**Table 4:** Normalized counts of platelet marker genes

**HD/TP**	***PPBP***	***TMSB4X***	***B2M***	***PF4***
HD_1	23,444	24,606	7,821	14,659
HD_2	42,122	78,497	27,720	21,999
HD_3	144,119	107,556	78,812	73,119
HD_4	23,279	61,195	11,420	17,009
HD_5	32,414	36,762	27,748	14,760
TP_1	68,510	172,492	53,738	61,160
TP_2	40,879	36,386	23,584	20,828
TP_3	44,637	99,445	51,791	34,089
TP_4	58,887	44,981	45,314	40,587
TP_5	197,738	157,380	126,643	111,286

### Patients platelet transcriptome is different from that of HDs

**Figure 1:**
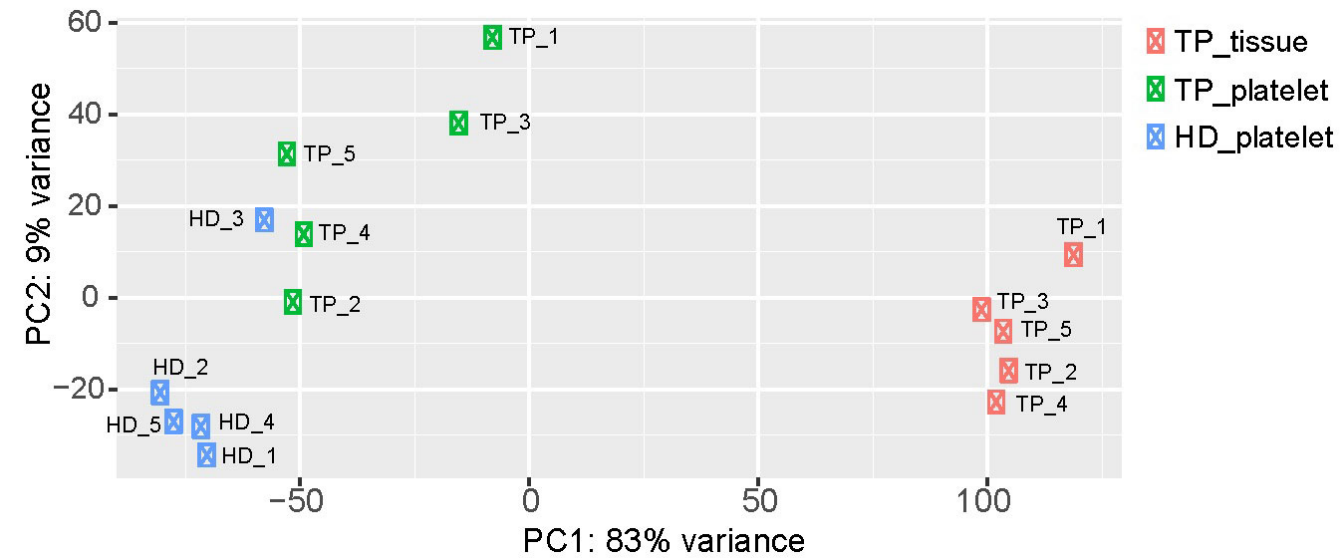
Two-dimensional visualization of the PCA. For each sample, the RNA expression profile, which was detected by RNA-seq, is displayed as one point in a two-dimensional space. The first two principal components capture 92% of the variability. Samples with similar expression profiles are grouped together. The three test groups (TP-tissue; TP-platelets; HD-platelets) separate from each other, suggesting differences in the expression profiles within the analyzed groups. PC: principal component

To get insights into the data set obtained by RNA-seq, PCA was carried out (Figure 1). The PCA revealed sample separation, which was in accordance with the biological groups (tumor patient-tumor tissue, tumor patient-platelets; HD-platelets). Since the PCA was computed based on the whole transcriptome profile, different RNA profiles in each group can be assumed. Moreover, the samples of the platelets derived from TPs scatter on both axes of the diagram (Figure 1) indicating that platelets of TPs contain a different cargo of RNA. Notably, the transcriptome of platelets of HD_3 is located within the group of platelets derived from TPs. In addition, the transcriptomes of platelets obtained from TP_1 and TP_3 separate from the samples of the other TPs and orient towards the tumor tissue samples.

In order to figure out the cause of the separation of the 2 groups of platelet samples, RNA-seq data were analyzed further. Comparing the RNA expression profiles of platelets derived from HDs and TPs resulted in 426 significantly differentially existing RNAs (FDR < 0.05; Table S1). Among these 426 mRNA, 406 were abundantly detected in the group of platelets derived from TPs, and interestingly, 20 mRNA were present in lower amounts in platelets derived from TPs in comparison to that of HDs.

By the use of the “Gene Ontology” platform, an enrichment analysis was carried out in order to get insights into the biological functions of identified differentially existing RNAs, which were assigned to a list of predefined biological terms [45, 46]. Term analyses revealed an association of detected RNA with squamous epithelium and keratinization. Likewise, RNA was also associated with terms of cell adhesion, signal transduction, or tumor-specific properties, such as proliferation, angiogenesis, and migration. Based on the information extracted from “Uniprot” and “NCBI Gene”, the 426 highly abundantly detected RNAs were further manually categorized, which confirmed this analysis: 49 RNAs encode genes that are characteristically expressed in epithelial cells and could have been transmitted by epithelial tumor cells (Table 5).

**Table 5:** Overview of genes with epithelial association

**Term**	**Genes**
Epithelial differentiation	*ZNF750* [49], *SFN* [50], *FAT2* [51, 52], *SBSN* [53], *GRHL1* [54], *GRHL2* [55], *TGM3* [56]
Epithelial cell adhesion	*EPCAM* [57], *DSP* [58], *CLDN4* [59], *DSG3* [60], *PKP1* [61], *DSC3* [60], *DSG2* [60], *CLDN1* [59], *CDH1* [62], *LAD1* [63], *CXADR* [64], *PKP3* [61], *F11R* [65], *PERP* [66]
Cytokeratin	*KRT5* [67], *KRT6a* [67], *KRT6b* [67], *KRT14* [67], *KRT17* [67], *KRT15* [67], *KRT75* [67], *KRT80* [67]
Keratinization	*PPL* [68], *BNC1* [69], *CNFN* [70], *KAZN* [71], *SPRR1A* [72], *SPRR2A* [72], *SPRR2B* [72], *SPRR2G* [72], *SPRR2F* [72], *SPRR2E* [72], *SERPINB13* [73], *KRTDAP* [74], *DMKN* [75], *EVPL* [76], *IRF6* [77], *FERMT1* [78]
Other epithelial characteristics	*ESRP1* [62], *ESRP2* [62], *LCN2* [79], *CerS3* [80]

Among the 426 differentially existing RNAs, 49 RNAs were found encoding genes characteristically expressed by epithelial cells. *SFN*: stratifin; *SBSN*: suprabasin; *GRHL1*: grainyhead like transcription factor 1; *TGM3*: transglutaminase 3; *EPCAM*: epithelial cell adhesion molecule; *DSP*: desmoplakin; *CLDN4*: claudin 4; *PKP1*: plakophilin 1; *DSC3*: desmocollin 3; *LAD1*: ladinin 1; *CXADR*: CXADR Ig-like cell adhesion molecule; *F11R*: F11 receptor; *PERP*: p53 apoptosis effector related to PMP22; *PPL*: periplakin; *BNC1*: basonuclin 1; *CNFN*: cornifelin; *KAZN*: kazrin; *SPRR1A:* small proline rich protein 1A; *SERPINB13*: serpin family B member 13; *KRTDAP*: keratinocyte differentiation associated protein; *DMKN*: dermokine; *EVPL*: envoplakin; *IRF6*: interferon regulatory factor 6; *FERMT1*: fermitin family member 1; *ESRP1*: epithelial splicing regulatory protein 1; *LCN2*: lipocalin 2; *CerS3*: ceramide synthase 3

In addition to epithelial markers, a great part of differentially existing RNAs code for genes associated with the hallmarks of cancer (Table 6) [81].

**Table 6:** Selection of significantly differentially expressed genes that correspond to the characteristics of tumors

**Genes**	**Function in tumor cells**	**Log_2_ fold change**	***P* adjusted**	**Reference**
*WNT5a*	Oncogen and tumor suppressor, associated with invasion, proliferation, and EMT	5.711	0.001	[82]
*MMP3*	Associated with metastasis	5.314	0.002	[83]
*SOX2*	Associated with tumor growth and metastasis	5.232	0.005	[84]
*TMPRSS4*	Associated with tumor proliferation and metastasis	5.153	0.002	[85]
*FOXE1*	Associated with tumor growth	4.949	0.020	[86]
*FOXQ1*	Associated with metastasis	4.882	0.005	[87]
*TNC*	Associated with metastasis and EMT	4.653	0.002	[88]
*TBX2*	Associated with tumor proliferation	4.508	0.007	[89]
*CRYAB*	Antiapoptotic factor	4.474	0.007	[90]
*MMP2*	Associated with metastasis	4.249	0.002	[83]
*PTHLH*	Associated with tumor growth	4.244	0.041	[91]
*EMP2*	Associated with angiogenesis	4.211	0.001	[92]
*CCN1*	Associated with tumor growth	4.203	0.006	[93]
*SPP1*	Associated with tumor growth and metastasis	4.196	0.018	[94]
*GPNMB*	Associated with metastasis	4.064	0.003	[95]
*ANO1*	Associated with metastasis	4.028	0.014	[96]
*MMP12*	Associated with metastasis	3.952	0.083	[83]
*YAP1*	Associated with tumor growth	3.870	0.001	[97]
*MMP13*	Associated with metastasis	3.832	0.077	[83]
*SNAI2*	Associated with EMT and metastasis	3.813	0.012	[98]

EMT: epithelial mesenchymal transition; *MMP3*: matrix metalloproteinase 3; *SOX2*: SRY-box transcription factor 2; *TMPRSS4*: transmembrane serine protease 4; *FOXE1*: forkhead box E1; *TNC*: tenascin C; *TBX2*: T-box 2; *CRYAB*: crystallin alpha B; *PTHLH*: parathyroid hormone like hormone; *EMP2*: epithelial membrane protein 2; *CCN1*: cellular communication network factor 1; *SPP1*: secreted phosphoprotein 1; *GPNMB*: glycoprotein nmb; *ANO1*: anoctamine 1; *YAP1*: Yes associated protein 1; *SNAI2*: snail family transcriptional repressor 2

Among these genes, MMPs, transcription factors, anti-apoptotic factors, and signaling proteins were identified. Some of these genes are even associated with a characteristic role in the tumorigenesis of head and neck carcinoma (like *SOX2* [84], *CRYAB* [90], and *PTHLH* [91]). Interestingly, 41 non-coding RNAs were significantly enriched. Two-thirds of these encode long non-coding RNAs.

**Figure 2:**
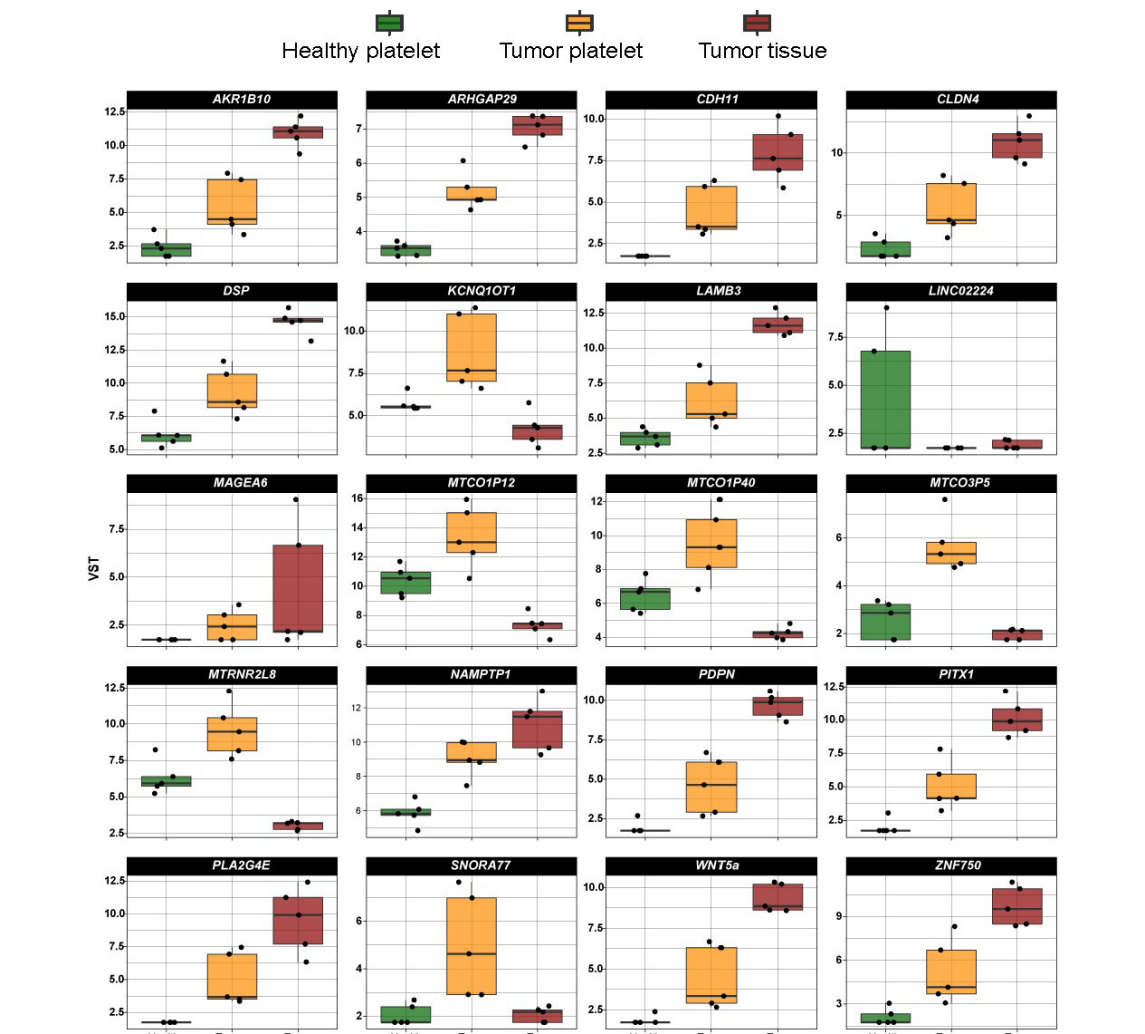
Expression profile of the top 20 differentially existing mRNA comparing platelets of HD and platelets of TPs in all test groups (TP-tumor tissue; TP-platelets; HD-platelets). Presented are box-plots. Each dot represents the value of 1 sample. The minimum, the lower quartile, the upper quartile and the maximum are shown. Depicted lines within the boxes represent the median. VST: variance stabilizing transformation; *ARK1B10*: aldo-keto reductase family 1 member B10; *ARHGAP29*: Rho GTPase activating protein 29; *LAMB3*: laminin subunit beta 3; *LINCO2224*: long intergenic non-protein coding RNA 2224; *MTRN2L8*: MT-RNR2 like 8 (pseudogene); *NAMPTP1*: nicotinamide phosphoribosyltransferase pseudogene 1; *PITX1*: paired like homeodomain 1; *SNORA77*: small nucleolar RNA, H/ACA box 77

A comparison of the level of mRNA existing in platelets from an HD, in TPs’ platelets and tumor tissue (Figure 2) revealed in some cases that the increase in platelet mRNA coding for certain genes is unlikely caused by the transfer of tumor-derived mRNA into platelets since the level of tumor RNA expression is low. This concerns for example the mRNA KCNQ1 opposite strand/antisense transcript 1 (*KCNQ1OT1*), MT-CO1 pseudogene 12 (*MTCO1P12*), *MTCO1P35*, and *MTCO1P40*. Therefore, genes abundantly existing in platelets from TPs compared to healthy platelets and present in tumor tissue were chosen for our further analyses. In doing so, RNA coding for 254 genes were identified, which will be further called “tumor-related mRNA” or “tumor-related genes” (Table S1–3).

**Figure 3:**
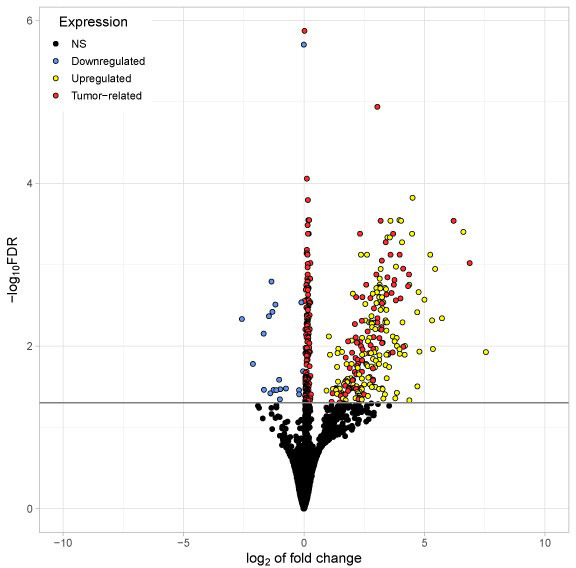
Volcano plot visualizing all significantly differentially existing RNAs detected in platelets of TPs *vs.* platelets of HDs. The x-axis represents the log_2_ fold change. The y-axis represents the –log_10_ of the adjusted *P* values (FDR). The horizontal line indicates the FDR threshold of 0.05. RNA shown in blue (20) appeared reduced in platelets from TPs compared to healthy. All other genes are upregulated in tumor platelets. Genes shown in red (254) are considered to be tumor-related (abundantly found in both platelets and tumor tissue from TPs). Genes shown in yellow (152) are abundantly found in platelets from TPs but not highly expressed in tumor tissue. NS: not significant

The volcano (Figure 3) plot illustrates all significantly differentially existing RNAs including “tumor-related mRNA” detected in platelets of TPs *vs.* platelets of HDs. Differentially existing and “tumor-related mRNA” are marked in red. Interestingly, the mRNA abundantly detected in tumor platelets lies in the region with a high log_2_-fold change of expression.

**Figure 4:**
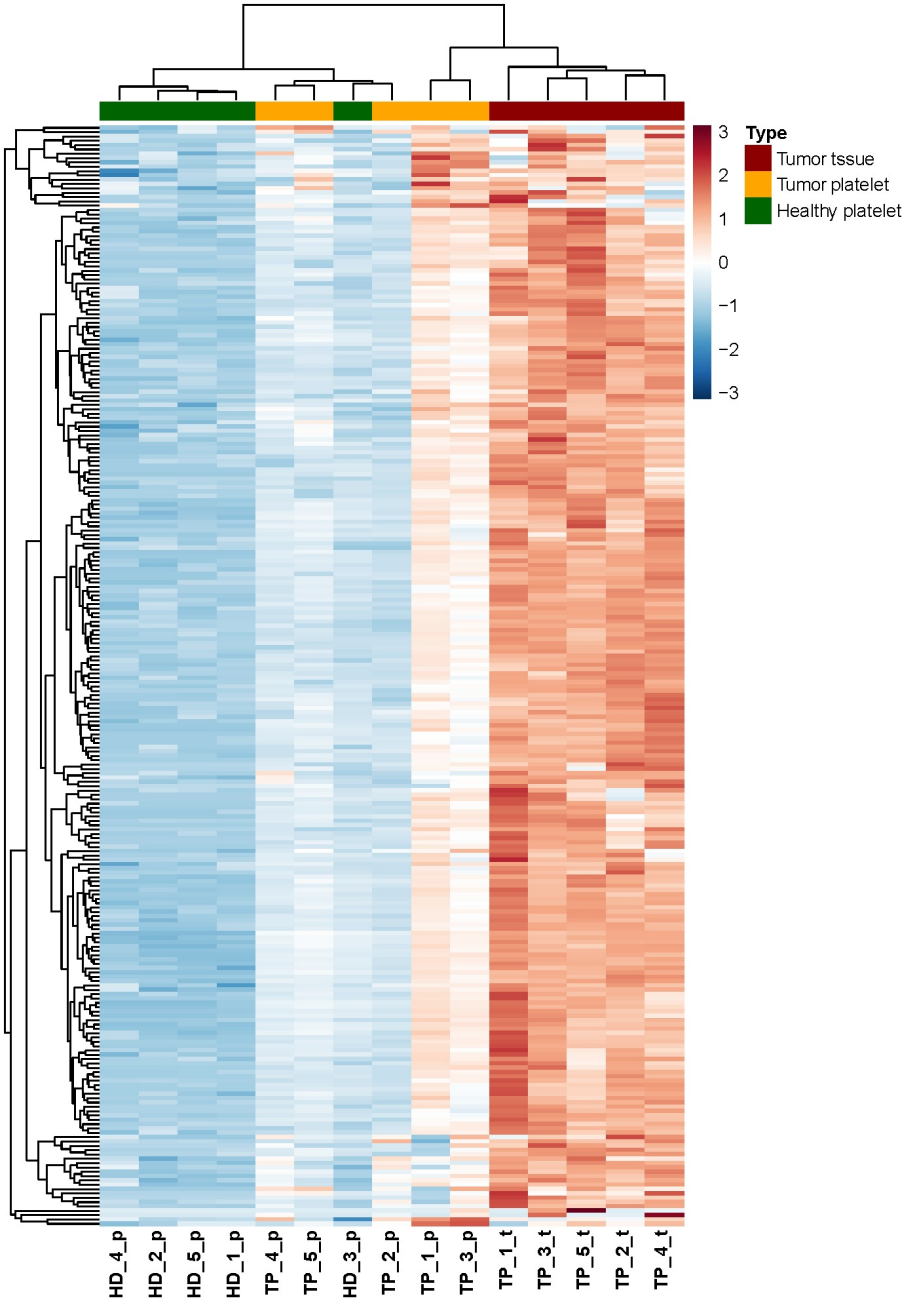
Heatmap of RNA abundance showing the 254 tumor-related and platelet-derived, significantly enriched RNA between the three groups (TP-platelets, HD-platelets, and TP-tumor tissue). HD_‘number’_p: HD_‘number’_platelets; TP_‘number’_p: tumor patient_‘number’_platelets; TP_‘number’_t: tumor patient_‘number’_tumor tissue

In order to analyze the expression profile of “tumor-related mRNA” even more, a heatmap using hierarchical clustering has been computed, showing the expression of all 254 tumor-related genes (Figure 4). A dendrogram shows a cluster built by the gene profiles of HD_1, HD_2, HD_4, and HD_5. The samples HD_1 and HD_5 bear the greatest similarities concerning their expression profiles. The profiles of the three TPs TP_4, TP_5, and TP_2 are also of high similarity. Notably, as already revealed by PCA (Figure 1), the profile of the HD_3 is close to that of the TPs. Apart from that patient, a separation of TPs and HDs is visible. In the samples, TP_3 and TP_1, almost every existing RNA, are higher expressed than in all the other TP samples. Also, a clear gradient increase in expression can be observed across the 3 groups.

### In search of platelet-associated predictive markers allowing the diagnosis of HNSCC

**Figure 5:**
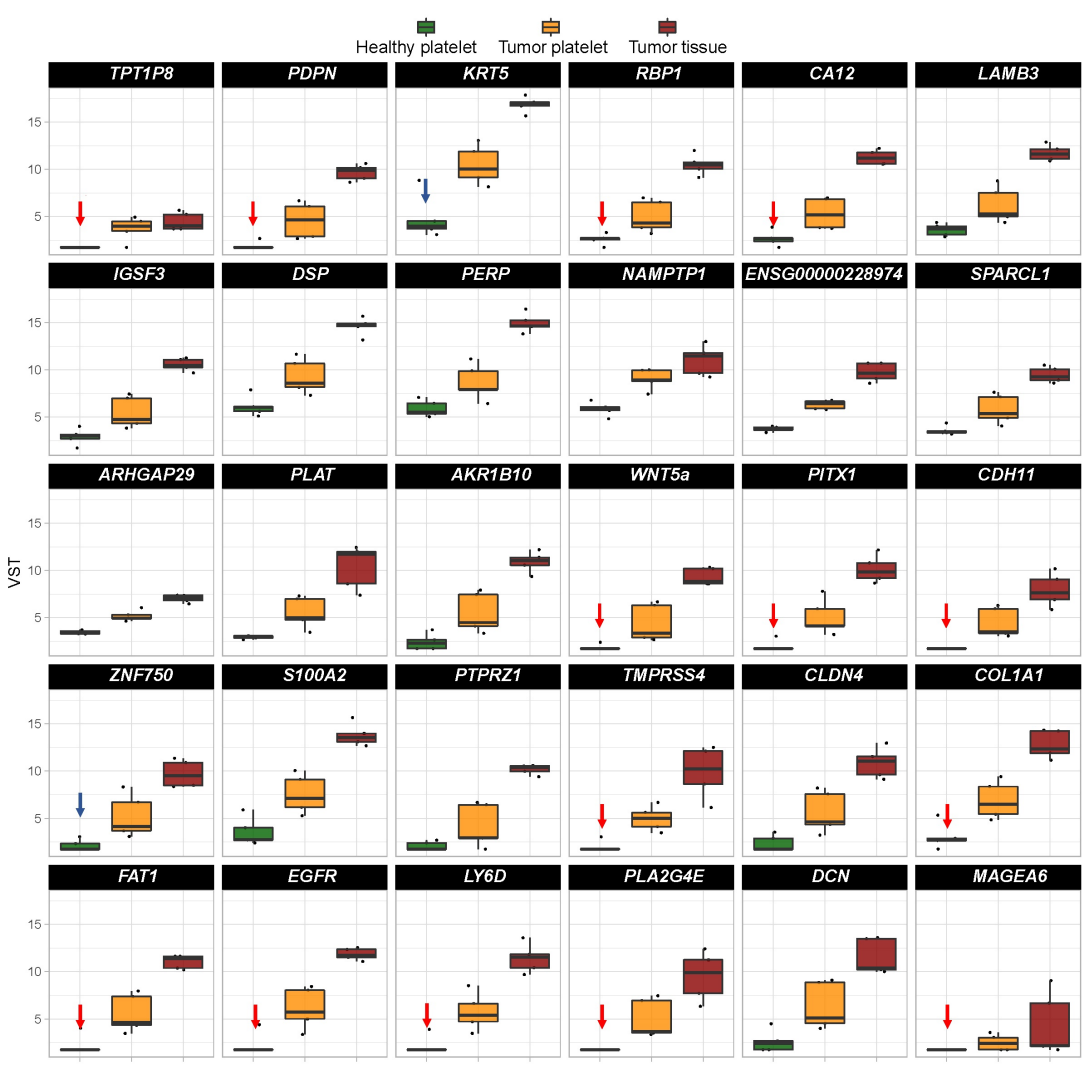
Boxplots revealing the expression profile of the top 30 differentially existing “tumor-related mRNA” comparing platelets of HDs and platelets of TPs in all test groups (TP-tumor tissue; TP-platelets; HD-platelets). The genes are sorted by absolute log_2_ fold change in descending order. Presented are box-plots. Each dot represents the value of 1 sample. The minimum, the lower quartile, the upper quartile and the maximum are shown. Depicted lines within the boxes represent the median. Selection of mRNA with a very low abundance in platelets of HDs (*n* = 14; indicated with red arrows) and of two additional mRNA based on high log_2_ fold change and their specific function in cancer (*n* = 2; indicated with blue arrows) for identification of platelet-based HNSCC-specific biomarkers. *TPT1P8*: TPT1 pseudogene 8; *IGSF3*: immunoglobulin superfamily member 3; *ENSG000002289*: RNA gene which is affiliated with the long non-coding RNA (lncRNA) class; *SPARCL1*: SPARC like 1; *PLAT*: plasminogen activator, tissue type; *S100A2*: S100 calcium binding protein A2; *PTPRZ1*: protein tyrosine phosphatase receptor type Z1; *PLAZG4E*: phospholipase A2 group IVE; DCN: decorin

In order to identify a biomarker set as a potential diagnostic tool, on the top 30 “tumor-related” differential existing mRNA in platelets of TPs *vs.* healthy controls was focused (Figure 5). In order to select a robust set of mRNAs allowing the prediction of a tumor in the head and neck region with a very low error rate, those mRNA with a very low abundance in platelets of HDs were selected (*n* = 14; indicated with red arrows in Figure 5). Additionally, two additional mRNA based on high log_2_ fold change and their specific function in cancer of epithelial origin, KRT5 [67, 99] and ZNF750 [100–102] (indicated with blue arrows in Figure 5) were selected.

**Figure 6:**
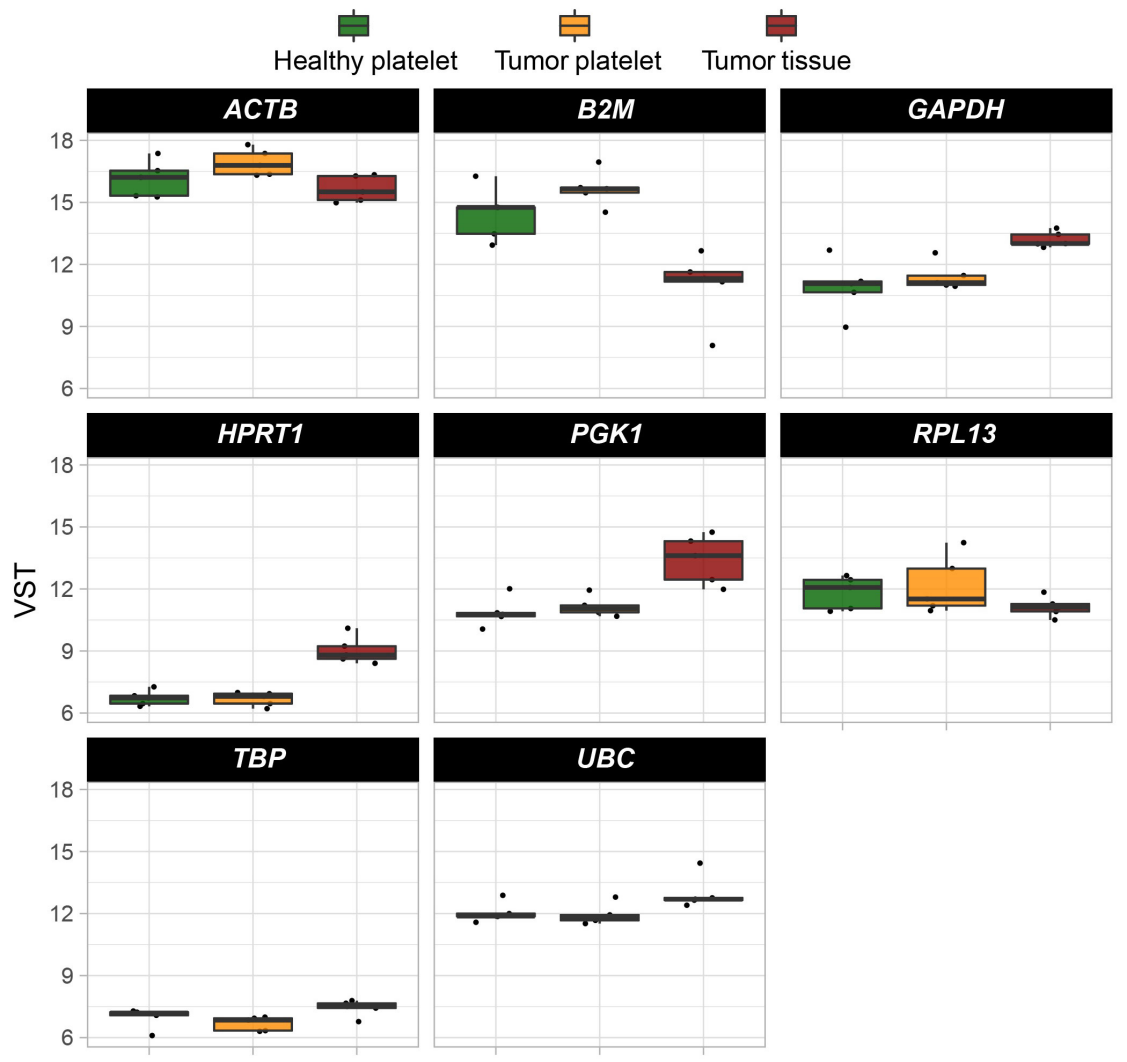
Boxplots reveal the expression profile of eight selected housekeeping genes. Their abundance was estimated by comparing all test groups (TP-tumor tissue; TP-platelets; HD-platelets). Presented are box-plots. Each dot represents the value of 1 sample. The minimum, the lower quartile, the upper quartile and the maximum are shown. Depicted lines within the boxes represent the median. *ACTB*: actin beta; *RPL13*: ribosomal protein L13; *TBP*: TATA binding protein

The abundance of these mRNA was determined in a second cohort of HDs and TPs. The characteristics of the analyzed cohort here are depicted in Tables 4–6. In addition, the level of existing mRNA in a set of housekeeping genes in order to exclude those housekeepers showing variation in the platelet-derived mRNA abundance between HDs and TPs were checked. Thus, for further analyses and data normalization, *GAPDH*, *UBC*, *HPRT*, and *PGK1* have been chosen (Figure 6).

**Figure 7:**
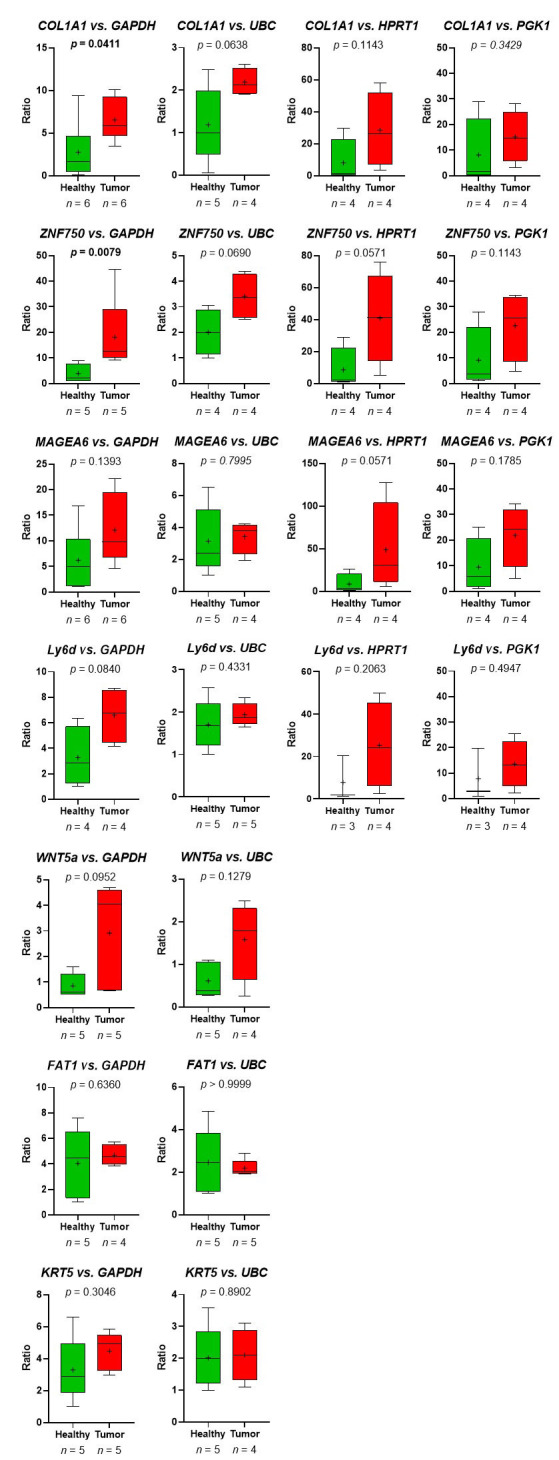
The relative abundance of target mRNA coding for *COL1A1*, *ZNF50*, *MAGEA6*, *Ly6D*, *WNT5a*, *FAT1*, and *KRT5* estimated relative to the expression of housekeeping genes *GAPDH*, *UBC*, *HPRT1*, and *GGK1* in platelets of HDs *vs.* TPs. Depicted lines within the boxes represent the median, and “+” represents the arithmetical mean. When significant differences were observed, the *P* value is highlighted in bold. The *P* value was calculated according to Mann-Whitney-*U* tets or Welch’s *t*-test depending on the data’s normal distribution

First, primer pairs, which were designed using NCBI primer blast (Table 3), were tested using human Universal Reference RNA (Thermo Fisher) as well as the HNSCC cell lines UDSCC1 or UDSCC5, for which in a previous RNA-seq project the expression of genes of interest was detected. For 12 out of 16 genes, primer pairs useful for quantitative reverse transcription (qRT)-PCR could be established. For *PLA2G4E*, *TMPRSS4*, *PITX1*, and *TPT1P8*, two to four primer pairs were tested. Unfortunately, none of these primer pairs amplified a fragment of the expected size. For 5 out of 12 primer pairs, amplifying *EGFR*, *PDPN*, *CDH11*, *CA12*, and *RBP1*, no amplicon could be detected analyzing platelet-derived mRNA. However, using the left 7 primer pairs amplifying *COL1A1*, *ZNF750*, *MAGEA6*, *Ly6D*, *WNT5a*, *FAT1*, and *KRT5* in all cases, except *FAT1*, a gain of predicted mRNA in platelets of TPs *vs.* platelets of HDs was observed (Figure 7).

**Figure 8:**
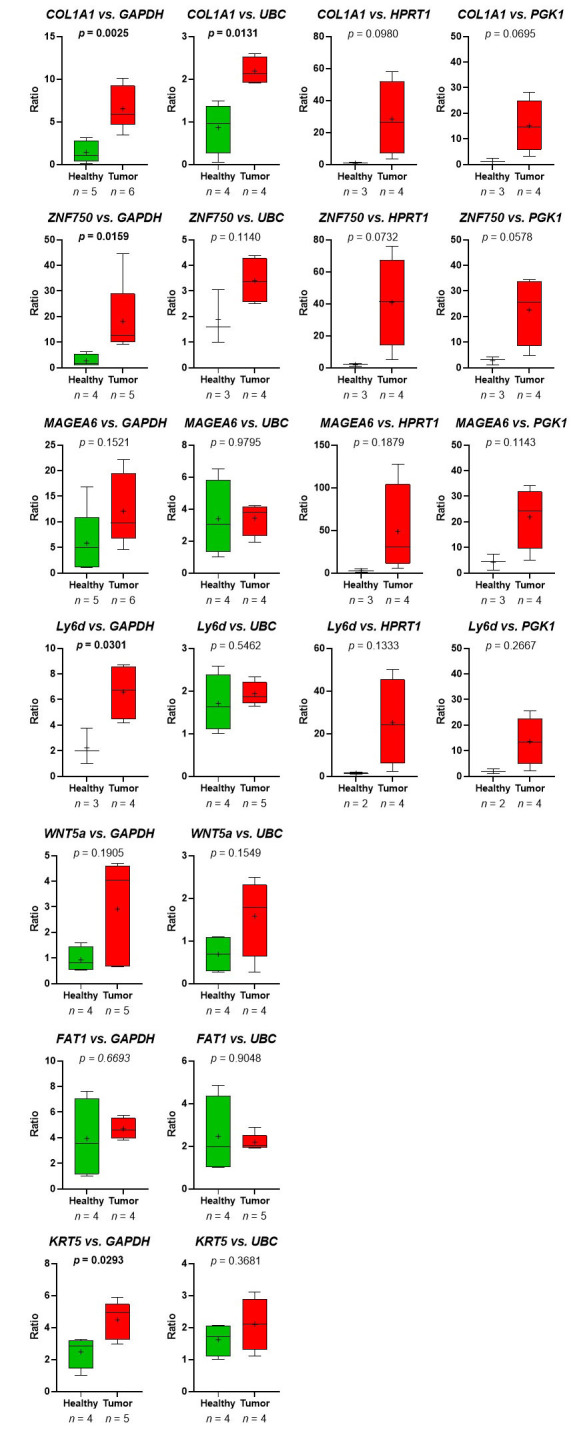
Presented are the relative abundance of target mRNA coding for *COL1A1*, *ZNF50*, *MAGEA6*, *Ly6D*, *WNT5a*, *FAT1*, and *KRT5* estimated relative to the expression of housekeeping genes *GAPDH*, *UBC*, *HPRT1*, and *GGK1* in platelets of HDs *vs.* TPs by boxplots. Here, the young HD (HD_12) with chronic tonsillitis and a smoking history was excluded from the HD group. Depicted lines within the boxes represent the median, and “+” represents the arithmetical mean. When significant differences were observed, the *P* value is indicated in bold. The *P* value was calculated according to Mann-Whitney-*U* or Welch’s *t*-test depending on the data’s normal distribution

Thus, the RNA-seq-data could be verified. Despite the fact, that in the group of HDs, one sample (HD_12; Table 2) behaved like a platelet mRNA derived from a TP, significant differences in *COL1A1* and *ZNF750* mRNA content in platelets of TPs *vs.* healthy controls were revealed (Figure 7). Static tests were applied again excluding the data derived from person HD_12. In this case, in 4 out of 7 analyzed genes, significant differences between mRNA abundance in platelets of HDs *vs.* TPs were revealed using GAPDH for normalization (Figure 8). In some cases, the significance level could not reach caused by a low sample number rather than a high sample variation. Unfortunately, due to the limited amount of mRNA extracted from platelets, not all TPs, as well as healthy individuals could be included in all analyses. For the same reason, not all predefined housekeeping mRNA was used for normalization. Of note, there are high variations in the abundance of analyzed mRNA in the TP’s group, independent of the tumor stage (data not shown).

Taken together, in this pilot study differentially existing mRNA profiles between platelets of HDs *vs.* HNSCC patients were identified. Moreover, the basis for a biomarker-panel development useful for diagnosis and possibly progress or therapy control of head and neck tumors using liquid biopsy-obtained platelets was created.

## Discussion

The current knowledge of thrombocyte biology, especially in the context of cancer, still leaves many unanswered questions. In addition, the mechanism responsible for changes in the RNA profile in platelets of TPs is not completely understood. However, the quite big array of publications over recent years describing differences in mRNA expression between HDs and patients of different tumor entities is not to be neglected. Also, the comparative analysis of the RNA profiles of platelets of HDs *vs.* HNSCC TPs revealed 426 differentially existing RNA. Among them, 254 “tumor-related RNA”, coding for epithelial markers, and, importantly, for tumor-associated genes were identified. Particularly, cytokeratin 5 and 14 are characteristic elements of the squamous epithelium [67], the origin of HNSCC. Additionally, the presence of RNA, which encodes oncogenes or tumor suppressor genes that are characteristically expressed in HNSCC, like *SOX2* [84], *CRYAB* [90], and *PTHLH* [91], further suggests transmission of tumor RNA into platelets [25, 81]. Therefore, the existence of epithelial and tumor marker RNA in TPs’ platelets may cause by the previous observations that tumor cells transfer their RNA into platelets [25].

Furthermore, 41 non-coding RNA forms were significantly differentially enriched in platelets derived from TPs *vs.* HD platelets, which could be explained by the fact that some of these forms are also poly-adenylated and therefore addressed by this approach [103]. Also, in the study of Best et al. [26], 20 differentially expressed non-coding RNAs were detected. The significantly altered existence of long non-coding RNA in platelets of TPs was also confirmed by Luo et al. [104]. In the present work, as well as in Best et al. [26], among others, the long intergenic non-protein coding RNA 152 (LINC00152) was abundantly found in TPs’ platelets, a long non-coding RNA expressed in many types of cancer, including oral squamous cell carcinoma [105, 106], acting as a potent oncogene involved in cancer cell proliferation, invasiveness, and motility.

Together, the presence of characteristics for cancer cells non-coding RNA as well as of RNA coding for epithelial and cancer-specific genes could indicate a transfer of RNA from nuclear tumor cells. However, these data should be interpreted with caution as the existence of platelet-coated CTC and an association of tumor-derived extracellular vesicles with platelets have been described [107, 108]. Therefore, contamination of platelet RNA with RNA from other tumor-derived sources cannot be excluded.

Despite the small cohort size, qRT-PCR results could validate significantly differentially existing mRNAs for 2 out of the 7 examined genes, namely *ZNF750* and *COL1A1*. These are very promising findings: whereas ZNF750 is a lineage-specific tumor suppressor in squamous cell carcinoma [49, 100–102], COL1A1 is associated with many tumor entities including oral and pancreatic cancer with prognostic relevance [109–111]. Additionally, in gastric cancer, COL1A1 is highly expressed and associated with cancer cell invasion, metastasis, and poor prognosis [112, 113]. COL1A is also a driver of oncogenesis in hepatocellular carcinoma [114]. Importantly, COL1A1 was recently identified as a candidate biomarker for HNSCC [99] and associated with cancer stem cell signature in HNSCC [115]. Thus, COL1A1 may serve as a possible diagnostic as well as a prognostic biomarker not only for HNSCC but also for other solid tumor entities. It, therefore, seems reasonable to implement the molecular COL1A1 characterization as a biomarker in the routine clinical/pathological molecular HNSCC diagnostic.

Although non-significant, many genes demonstrate a clear trend, where platelets of TPs show elevated mRNA levels compared to those of healthy individuals, this could be seen in almost all cases in varying degrees, except for FAT1. Reasons for non-significant results could be variations in individual patients or the small sample sizes, which is a limitation of the present study. Although sample size calculations according to Lin et al. [37] were performed before starting the here described pilot study, not all hypotheses could be checked with the estimated sample size. However, it is remarkable that, despite this limitation, the abundance of tumor-related RNA could be verified in a second, equally small cohort.

After taking a closer look at the individual results, two HDs appeared to behave like a TP (cohort 1: HD_3; cohort 2: HD_12), whose platelets were showing substantial “tumor-related mRNA” abundance. There was an attempt to acquire a more recent blood sample in order to investigate whether any malignancies are present now, but unfortunately, the donors have not agreed. While no further details on the health status of HD_3 is available, the HD HD_12 was a chronic tonsillitis patient, 22 years old of age at the time of the blood draw (5 years ago), with a tobacco and alcohol consumption history. Since the results obtained from platelet RNA of HD_12 were suspicious, it was hypothesized that this individual had a pathological condition influencing platelet physiology. This is likely since changes in gene expression profile, as well as platelet count in patients of chronic tonsillitis and other diseases, were observed [116–118].

However, based on an HNSCC-causing history in the case of HD_12, the possibility cannot be excluded, that with the method applied here, a pre-cancerogenic processes in that person classified as a HD was identified. To prove this assumption, prospective studies should be conducted analyzing platelet mRNA content routinely and the patients should be observed for years.

Additionally, one TP (TP_8) could be considered a HD. One could assume that with early tumor stage less tumor RNA can be taken up by platelets. However, this association turned out to be inconsistent in the here performed analyses. But certain drugs inhibit the function of platelets which could affect the interaction of tumor cells and platelets [119], and consequently the amount of transferred mRNA.

Statistical procedures exist to detect outliers [120]. However, the data presented here stem from a small sample size and most do not follow a normal distribution, so it was not possible to reliably apply these methods. However, in order to determine sample sizes for this pilot study we followed the suggestions of Lin et al. [37].

The first and fundamental question this work attempted to answer was whether a transfer of tumor RNA to platelets actually takes place since platelets from head and neck cancer patients have not been analyzed so far. And this study was able to answer this question positively using this small cohort. Additionally, applying this study design, a set of RNA that was specified as “tumor-related” in a second, again small cohort, was identified as at least enriched in patient platelets. Precisely because the data were collected using small cohorts, these findings show the great possible diagnostic potential of platelet analysis in head and neck cancer patients.

A strength of the present study is the inclusion of tumor RNA into the sequencing analyses and not just comparing platelets of TPs with that of HDs. Doing so, it was revealed that differentially existing RNA in platelets is not exclusively caused by the transfer from tumor to platelets. This observation has to consider for further studies on TEP. There are many factors influencing the platelet transcriptome even under non-pathological conditions. According to Simon et al. [121], the mRNA profiles of platelets even differ depending on the age of the patients, their ethnicity, or their gender. As already mentioned, different diseases can cause transcriptomic changes in platelets [116–118]. Moreover, there is a certain platelet transcriptome variance observed even in HDs, probably depending on the platelet subpopulation [122, 123]. All these factors could contribute to individual variations and to the initial observation of the present study that not all differentially existing RNA observed in platelets of TPs are characterized by a gain of RNA. Thus, there is an urgent need for robust biomarkers allowing the identification of defined diseases, including HNSCC.

Despite the limitations of this work, the presented results are very promising, because so far, there is a lack of effective screening options for the diagnostics and therapy monitoring of HNSCCs [124] and liquid biopsy poses a great potential for the future of cancer diagnostics. The results presented here provide a basis for a biomarker panel that shows a potential ability for platelets to distinguish between healthy and TPs in HNSCC tumors with a simple blood draw.

With the help of future research, the analysis of platelet RNA in head and neck TPs could generate a powerful method for monitoring tumorigenesis at any time and in real-time. As was suggested also for other cancer entities, tumor-specific therapies could be applied in a targeted manner and modified with respect to response rate or resistance development [6, 34, 125].

## Abbreviations

B2M: beta 2-microglobulin
*CDH11*: cadherin 11
*CLDN4*: claudin 4
COL1A1: collagen type I alpha 1 chain
*CRYAB*: crystallin alpha B
CTCs: circulating tumor cells
*DSP*: desmoplakin
EGFR: epidermal growth factor receptor
EMT: epithelial mesenchymal transition
*FAT1*: FAT atypical cadherin 1
FDR: false discovery rate
*GAPDH*: glyceraldehyde-3-phosphate dehydrogenase
HDs: healthy donors
HNSCC: head and neck squamous cell carcinoma
*HPRT1*: hypoxanthine phosphoribosyltransferase 1
HPV: human papilloma virus
*KRT5*: keratin 5
*Ly6D*: lymphocyte antigen 6 family member D
*MAGEA6*: MAGE family member A6
*MMP3*: matrix metalloproteinase 3
mRNA: messenger RNA
*MTCO1P12*: MT-CO1 pseudogene 12
NCBI: national center for biotechnology information
PCA: principal component analysis
PCR: polymerase chain reaction
*PDPN*: podoplanin
*PGK1*: phosphoglycerate kinase 1
*PITX1*: paired like homeodomain 1
*PLA2G4E*: phospholipase A2 group IVE
*PTHLH*: parathyroid hormone like hormone
py: pack years
*RBP1*: retinol binding protein 1
RNA-seq: RNA-sequencing
*SOX2*: SRY-box transcription factor 2
*SPRR1A*: small proline rich protein 1A
TEP: tumor-educated platelets
*TMPRSS4*: transmembrane serine protease 4
TPs: tumor patients
*UBC*: ubiquitin C
*WNT5a*: Wnt family member 5a
*ZNF750*: zinc finger protein 750

## Supplementary materials

The supplementary material for this article is available at: https://www.explorationpub.com/uploads/Article/file/1002143_sup_1.xlsx.

## Declarations

### Availability of data and materials

RNA-seq data are available from Gene Expression Omnibus (GEO, https://www.ncbi.nlm.nih.gov/geo/query/acc.cgi?acc=GSE156554) under Record ID GSE156554.

### Copyright

© The Author(s) 2023.
